# Prediction of blunt traumatic injuries and hospital admission based on history and physical exam

**DOI:** 10.1186/s13017-016-0099-9

**Published:** 2016-08-31

**Authors:** Alan L. Beal, Mark N. Ahrendt, Eric D. Irwin, John W. Lyng, Steven V. Turner, Christopher A. Beal, Matthew T. Byrnes, Greg A. Beilman

**Affiliations:** 1 North Memorial Medical Center, 3300 Oakdale Ave N, Robbinsdale, MN 55431 USA; 2 University of Minnesota, Minnesota, USA; 3 North Memorial Medical Center, Minnesota, USA

**Keywords:** Trauma, Injuries, Triage, Imaging

## Abstract

**Background:**

We evaluated the ability of experienced trauma surgeons to accurately predict specific blunt injuries, as well as patient disposition from the emergency department (ED), based only on the initial clinical evaluation and prior to any imaging studies. It would be hypothesized that experienced trauma surgeons’ initial clinical evaluation is accurate for excluding life-threatening blunt injuries and for appropriate admission triage decisions.

**Methods:**

Using only their history and physical exam, and prior to any imaging studies, three (3) experienced trauma surgeons, with a combined Level 1 trauma experience of over 50 years, predicted injuries in patients with an initial GCS (Glasgow Coma Score) of 14–15. Additionally, ED disposition (ICU, floor, discharge to home) was also predicted. These predictions were compared to actual patient dispositions and to blunt injuries documented at discharge.

**Results:**

A total of 101 patients with 92 blunt injuries were studied. 43/92 (46.7 %) injuries would have been missed by only performing an initial history and physical exam (“Missed injury”). A change in treatment, though often minor, was required in 19/43 (44.2 %) of the missed injuries. Only 1/43 (2.3 %) of these “missed injuries” (blunt aortic injury) required surgery. Sensitivity, specificity, and accuracy for injury prediction were 53.2, 95.9, and 92.3 % respectively. Positive and negative predictive values were 53.8 and 95.8 % respectively. Prediction of disposition from the ED was 77.8 % accurate. In 7/34 (20.6 %) patients, missed injuries led to changes in disposition. “Undertriage” occurred in 9/99 (9.1 %) patients (Predicted for floor but admitted to ICU). Additionally, 8/84 (9.5 %) patients predicted for floor admission were sent home from the ED; and 5/13 (38.5 %) patients predicted for ICU admission were actually sent to the floor after complete evaluations, giving an “overtriage” rate of 13/99 (13.1 %) patients.

**Conclusions:**

In a neurologically-intact group of trauma patients, experienced trauma surgeons would have missed 46.7 % of the actual injuries, based only on their history and physical exam. Once accurate diagnoses of injuries were completed, usually with the help of CT scans, admission dispositions changed in 20.6 % of patients. Treatment changes occurred in 44.2 % of the missed injuries, though usually minimal. Broad elimination of early imaging studies in alert, blunt trauma patients cannot be advocated.

## Background

Diagnostic accuracy and efficiency are important in the initial trauma evaluation. Goals also include limitation of patients’ time spent in the ED, compiling an accurate list of injuries, and making rapid, safe, disposition decisions; i.e.; operating room, ICU, floor or discharge to home. Early and aggressive imaging of the trauma patient, using plain films, ultrasound, and a wide variety of CT scans has become commonplace in the trauma evaluation. The yield of CT scans varies in blunt trauma victims, creating inconsistent recommendations for their use, especially in alert patients [1–3]. We returned to the basics of clinical medicine in this prospective study and evaluated the accuracy of the history and physical exam when carried out by experienced trauma surgeons on a group of awake patients. We also tried to predict the emergency department disposition of these patients, hoping to speed up their admission process.

## Methods and study design

The study was reviewed by the North Memorial IRB, and waiver of consent requirements was granted. The study was conducted by three trauma surgeons with similar levels of training and experience. All surgeons completed surgical residencies between 1988 and 1994, and have a combined experience of 56 years at our Level 1 trauma center. A total of 101 non-consecutive blunt trauma patients with a Glasgow Coma Score of 14–15 were evaluated over nine (9) months, prior to completion of any radiologic imaging. Specific injuries were predicted, based only on the history and physical exam. Patients were excluded if any imaging studies, including ultrasonography, had been completed prior to the trauma surgeons’ evaluation. Patients underwent collection of medical history and a physical examination, and specific injuries were then predicted in each of eleven (11) categories, using a standardized prediction worksheet. The patient’s emergency department disposition was also predicted and recorded prior to imaging studies; i.e., ICU, floor, or discharge to home. “Missed injuries” were defined as those not predicted by the trauma surgeon on the admission prediction worksheet, but eventually diagnosed during the hospitalization. Most patients had multiple imaging studies after predictions had been made. All missed injuries, incorrect diagnoses and incorrect patient dispositions were recorded. Any change in treatment plans were noted for each of the missed injuries.

Sensitivity, specificity, accuracy, as well as positive and negative predictive values was determined for all predicted injuries. The overall accuracy, “overtriage” and “undertriage” rates were determined for the predicted dispositions to the ICU, floor or discharge to home. A comparison of patients’ ages and ISS was made between the group with “missed injuries” and those without missed injuries, using a paired *t* test method. Those patients with a GCS of 14 were compared in the same two groups (“Missed injury vs no missed injury) with those with GCS of 15, using a two-tailed Fisher’s exact test.

## Results

Table 1 gives a breakdown of the number of patients seen and entered into the study by each surgeon, as well as the number of injuries diagnosed and “missed” by each of them. Table 2 gives basic demographics of the 101 blunt trauma patients.

**Table 1:** Injuries and missed injuries

Surgeon	# of Pts	Total # of injuries	# of Pts with no injuries	# of missed injuries
Surgeon A	18	17	7	9
Surgeon B	45	46	16	15
Surgeon C	38	29	15	19
Total	101	92	38	43

**Table 2:** Patient Demographics

Demographics	Data
Sex	Males: *N* = 70 Females: *N* = 31
Age	Mean: 38.1 Range: 3-88
ISS	Mean: 8.0 Range: 0-38
GCS Score	GCS of 15: *N* = 91 GCS of 14: *N* = 10

Table 3 gives details of the three combined surgeons’ accuracies in each of the eleven injury categories addressed on the prediction worksheet.

**Table 3:** Accuracy of Predicting Injuries with H/P

Injury	TP^1^	FP^2^	TN^3^	FN^4^	Sens^5^	Spec^6^	NPV^7^	PPV^8^	Accur^9^
Brain	3	6	86	6	33.3	93.5	93.5	33.3	88.1
Cervical Fracture	1	4	92	4	20.0	95.8	95.8	20.0	92.1
Rib Fractures	7	9	78	7	50.0	89.7	91.8	43.8	84.2
Pneumothorax	1	1	96	3	25.0	99.0	97.0	50.0	96.0
Solid organ injury	1	4	94	2	33.3	95.9	97.9	20.0	94.1
Pelvic Fracture	4	4	91	2	66.7	97.8	97.8	50.0	94.1
T/L Spine Fracture	3	4	85	8	27.2	95.5	91.4	42.9	87.1
Extremity Fracture	22	6	68	5	81.5	91.9	93.2	78.6	89.1
Clavicle Fracture	5	1	91	4	55.6	98.9	95.8	83.3	95.0
Vascular Injury	0	0	99	2	0	100	98.0	0	98.0
Spinal Cord Injury	2	3	96	0	100	96.7	100	40.0	97.0
Total	49	42	976	43	53.2	95.9	95.8	53.8	92.3

^1^ *TP* True positive, ^2^ *FP* False positive, ^3^ *TN* True negative, ^4^ *FN* False negative, ^5^ *Sens* Sensitivity, ^6^ *Spec* Specificity, ^7^ *NPV* Negative predictive value, ^8^ *PPV* Positive predictive value, ^9^ *Accur* Accuracy

Combined, the surgeons’ overall outcome in predicting injuries based on the initial history and physical exam includes a 53.2 sensitivity, 95.9 specificity, 95.8 negative predictive value, and a 53.8 % positive predictive value, for an overall accuracy of 92.3 %.

Results of the predictions for the disposition of the patients are shown in Table 4. A total of 9/84 (10.7 %) patients predicted for a floor admission were instead admitted to the ICU due to their missed injuries (“undertriage”). Also, 8/84 (9.5 %) patients predicted to go to the floor were actually able to go home after their evaluations, while 5/13 (38.5 %) predicted to go to the ICU were able to go to the floor, for an “overtriage” rate of 13/97 (13.4 %). Overall disposition accuracy was 77.8 %.

**Table 4:** Prediction of Patient Disposition

Predicted disposition	Actual disposition: floor	Actual disposition: ICU	Actual disposition: home
Floor	67	9	8
ICU	5	8	0
Home	0	0	2

A total of 43 “missed injuries” occurred in 34 patients. Nine patients each had two missed injuries. The patients with missed injuries had a range of ages from 11–88. The mean age of those patients with missed injuries was older than those with accurate diagnoses (42.8 vs 34.5; *p* < 0.03) There were only two missed injuries in the eight pediatric patients, a clavicle fracture in an 11 year old, and a minimal wrist fracture in a 14 year old.

A total of 6/34 (17.6 %) patients with missed injuries had a GCS of 14, while 4/67 (6.0 %) without missed injuries had GCS of 14. (*p* = 0.08) The mean injury severity score (ISS) of the group with missed injuries was 12.6, as compared to an ISS of 5.7 in those patients without missed injuries. (*p* < 0.0001) Surprisingly, only 4/34 (11.8 %) with missed injuries had elevated blood alcohol levels (BAL) at the time of the initial evaluation.

Overall, 22/97 (22.7 %) patients had a change in their disposition compared to that predicted. Two patients did not have predictions made at the initial evaluation. Change in admission disposition due to at least one missed injury occurred in 7/34 (20.6 %) patients.. Change in therapy occurred in 19/43 (44.2 %) missed injuries, though most of these treatment changes were modest; e.g., extremity splinting, frequent neurological exam; analgesia. Only one patient required surgery for an injury not predicted by the initial history and physical exam: a blunt thoracic aortic injury.

Table 5 lists the number of missed injuries by categories and whether there were changes in treatment of disposition based on missed injuries.

**Table 5:** Numbers of “Missed Injuries” by Type

Injury type	*N* = 43	+Change Rx	+Change disposition
Traumatic Brain Injuries	6	3	3
Cervical Spine Fractures	4	1	0
Ribs/Sternum	7	4	1
Pneumothoraces	3	0	1
Solid Organ Injuries	2	1	0
Pelvic Fractures	2	0	0
Spine Fractures	8	2	0
Extremity Fractures	5	4	0
Clavicle/Scapular Fractures	4	2	0
Vascular	2	2	2

Only 1/6 of the missed traumatic brain injuries would be considered significant. None of the cervical spine fractures were considered serious, with only one needing a cervical collar. Two of 3 liver injuries were not predicted and both of these patients had short ICU admissions and successful non-operative management. Both of the “missed” pelvic fractures were minor. The surgeons did not predict 8/12 of the thoracolumbar fractures, but none of these fractures required surgery, and only 2 required orthotics. All of the “missed” extremity, clavicular and scapular fractures were modest; none required surgery. Both of the vascular injuries were not suspected on the history and physical. The unilateral vertebral artery injury was treated with anti-platelet therapy. The blunt thoracic aortic injury required an endovascular stent graft.

## Discussion

The history and physical exam can serve an important role in most trauma work-ups. Advanced Trauma and Life Support (ATLS) programs emphasize the use of a history and exam during both the primary and secondary surveys. Treatment of suspected life-threatening injuries can occur based only on the physical exam [4].

For this study, we challenged our most experienced trauma surgeons to prospectively predict injuries, as well as the patients’ emergency department (ED) disposition, prior to any imaging studies being completed. Evaluating a group of alert trauma patients (GCS 14–15) and knowing the accuracy of our predictions is a first step in potentially reducing the number of imaging studies, while decreasing patient time spent in the ED. In our study, however, 43/92 (46.7 %) injuries would have been missed if only the history and exam had been used for initial definitive diagnoses (“Missed injury”).

The reasons for our high missed injury rate are not clear. By choosing our most experienced surgeons, the impact of the inexperience factor was reduced. Complacence or inattention to detail in the history and physicals may have occurred despite their significant experience. In our institution, the trauma surgeons do not have regular house staff support and remain the frontline decision-makers for our trauma patients. In the modern trauma evaluation, ongoing reliance on imaging studies, such as CT scans, certainly could lead to less focus and concentration on the history and physical exam and erode these clinical skills.

We also identified poor “under/over”-triage rates when relying on the history and physical exam. Since our injury prediction accuracy was unacceptably low, this was likely the major cause for the undertriage rate for patient dispositions.

The injury severity score (ISS) was higher in the 34 patients with missed injuries (12.6 vs 5.7; *p* < .0001). This suggests that the unidentified injuries, or the associated pain, could have been a confounding factor in accurately assessing the extent of injury. The ISS is determined retrospectively by the trauma registrars and thus would not have changed based on our definition of a “missed injury”.

The Glasgow Coma Score (GCS) of the two groups (missed injuries vs. no missed injuries) was not significantly different (14.8 vs 14.7). However, 6/34 (17.6 %) patients with missed injuries had a GCS of 14, while only 2/67 (3.0 %) patients without missed injuries had a GCS of 14. (*p* = 0.08). Though not significant, the trend here between differences in mental status could have played a role in the overall accuracy of our history and physical exams.

The mean age of those patients with “missed injuries” was older than those with accurate diagnoses (42.8 vs 34.5: *p* < 0.03). It is not unusual for elderly patients to have abnormal pain thresholds in a variety of clinical scenarios. This could have had an effect on the history and physical exams of our patients.

It did not appear that alcohol played an important role in our diagnoses of injuries. A total of 14/50 (28 %) patients in the group without missed injuries had elevated blood alcohol levels (BAL). Only 4/35 (11.4 %) of patients in the group with “missed injuries” had elevated BAL (*p* = 0.10).

The study was originally designed to have a wider range of clinicians (medical students, surgical interns and critical care fellows) also make predictions in order to gather data from clinicians with a more varied range experiences however their rotations were relatively short and the number of evaluations completed by these clinicians was very small. Given the limited number of surgeons’ experiences within this study, it is possible that the accuracy of the predictions cannot be extrapolated to other trauma centers or surgeons. Results from this study indicate that a full evaluation, including imaging, in trauma patients will provide the most beneficial care plan, however further investigations are required to confirm these findings.

A number of studies have addressed the diagnostic accuracy of the history/physical in a variety of traumatic injuries. None have used a small, consistent and experienced group of clinicians, as we did in this study. Many have employed retrospective chart reviews, rather than a prospective approach.

Hoping to reduce the number of CT scans in blunt trauma patients, Tillou, Cryer and colleagues would have missed almost 17 % of injuries with use of their initial clinical evaluation [5]. Even in awake patients with a normal exam and stable hemodynamics, Salim et al. found “clinically significant findings” in 3.5 % of head CT's, 5.1 % of cervical CT’s, 7.1 % of abdominal CT’s and 19.6 % of chest CT scans. These findings changed patient management in 19 % of the patients [6].

Previous studies of traumatic brain injuries report up to a 20 % rate of abnormal head CT’s and a 5 % need for craniotomy, even with a normal clinical exam [7–9]. “The Canadian head CT rule” and the “New Orleans criteria” remain the best predictors of clinically-significant brain injuries in alert patients [10–12].

Clinical criteria to rule out cervical spine injuries have been evaluated. The National Emergency Radiologic Utilization Study (NEXUS) included over 34,000 patients in 21 centers, while the “Canadian C spine rule” prospectively developed clinical criteria to accurately rule out cervical injuries [13, 14]. These studies were the foundation for other more recent recommendations to help reduce the number of imaging studies needed, while simplifying the cervical evaluations in both adults and children [15–19].

The history and exam can be quite accurate for diagnoses in blunt chest injuries in both adults and children, arguing for fewer imaging studies [20–24]. Blunt abdominal trauma diagnoses can be challenging using only the history/physical. Patients with subjective symptoms and positive physical findings, such as bruising and tenderness, will have intra-abdominal injuries in only about 20 % of cases [25, 26]. On the other hand, the incidence of actual injuries with a negative exam is also reported to be up to 20 % [4, 25–32]. Other factors such as distracting injuries, low GCS and alcohol intoxication can affect the accuracy of the physical exam [26, 30, 33]. Physical exam in children has been shown to be more sensitive, but is still challenging without the support of other modalities such as ultrasound [34, 35].

Bedside clinical assessment for pelvic fractures can be sensitive in the alert patient [36]. False negative exams are present in 1-7 % of patients with the appropriate mechanism of injury. Physical exam can sometimes be more sensitive than plain x-rays [37, 38].

An in-depth review of the accuracy of the history/physical in diagnoses of thoracolumbar fractures found conflicting results [39]. Several reports support the premise that the lack of symptoms and tenderness predicts a very low risk for fractures [40, 41]. A prospective, predictive study by Holmes and colleagues supports these findings in alert patients [42]. Diagnostic guidelines for thoracolumbar spine evaluations have been established by the Eastern Association for Surgery of Trauma (EAST) [43]. On the other hand, 20-50 % of these fractures have been reported to have no symptoms or physical findings, even in alert patients [44–46].

Musculoskeletal injuries historically have been the most commonly missed traumatic injuries [47, 48]. The incidence of missed injuries or delayed diagnoses of musculoskeletal trauma has been reported to be from 1.3-39 %, with the higher rates seen in the more severely injured, and especially in those with altered mental status [48–51]. More than 20 % of these missed injuries can be clinically significant [52]. The usefulness of the clinical exam in diagnosis of musculoskeletal trauma has not been widely studied, and available data are mostly from studies of low energy, isolated injuries, often seen in ambulatory patients; e.g., elbow, wrist, hand [53–56].

While a great deal has been written about the evaluation and management of penetrating vascular injuries, blunt vascular trauma has been less well-studied. Blunt arterial injuries comprise only about 20 % of arterial trauma and can present with minimal clinical findings [57]. The mechanism or pattern of injury may be the only factor to make one suspicious for arterial injury. Blunt thoracic aortic injuries rarely have a blood pressure differential between the arms and legs. Even CXR’s are normal in 7.3-23 % of those with blunt thoracic aortic injuries [57]. A “traumatic aortic injury score” (TRAINS) has been reported, but relies more on the chest x-ray and the diagnosis of other associated injuries, rather than on the history and exam [58].

Clinical risk factors for a blunt carotid or vertebral artery injury were recently reported in a Western Trauma Association critical decision paper [59]. Unfortunately, up to 20 % of patients with such injuries have none of these risk factors [60]. Emphasizing the importance of timely and accurate diagnoses, the EAST group published practice management guidelines for blunt cerebrovascular injuries, and cite an 80 % morbidity and 40 % mortality rate if neurologic symptoms develop from these injuries [61].

## Conclusion

If only a history and physical exam is used for diagnosis in an alert group of trauma patients (GCS 14–15), experienced trauma surgeons at our hospital missed 46.7 % of their injuries. The reasons for these inaccurate clinical predictions are not clear, though the average injury severity score was higher and the age of the patients greater in those with missed injuries. This same approach to predicting a trauma patient’s hospital disposition was 77.8 % accurate, with 9.1 % of patients being “undertriaged” to the floor. Due to these results, though many of the “missed” injuries were minor and often did not require a change in treatment, we cannot advocate a broad elimination of early imaging studies, even in alert trauma patients.

## Abbreviations

BAL: Blood alcohol level
CT: Computed tomography
CXR: Chest X-ray
EAST: Eastern association for the surgery of trauma
ED: Emergency Department
GCS: Glascow coma score
ICU: Intensive care unit
ISS: Injury severity score
NEXUS: National emergency radiologic utilization study
TRAINS: Traumatic aortic injury score
